# Police involvement, characteristics and outcomes of place of safety referrals in the Scottish Highlands

**DOI:** 10.1192/bjb.2020.13

**Published:** 2020-12

**Authors:** Struan Simpson, Jude Eze

**Affiliations:** 1 The Phoenix Centre, Raigmore Hospital, Inverness, UK; 2 Epidemiology Research Unit (Inverness Campus), Scotland's Rural College, Inverness, UK

**Keywords:** Suicide, risk assessment, self-harm, service users, psychiatry and law

## Abstract

**Aims and method:**

To characterise police involvement with those detained under place of safety legislation and determine factors associated with admission to hospital. Place of safety referrals over a 1-year period were identified retrospectively and evaluated.

**Results:**

Place of safety legislation is generally used with regard to concerns about suicide. Individuals are often removed from high-risk areas and referrals to police are frequently initiated by individuals themselves. A diagnosis of mental illness or personality disorder predicted hospital admission. Presence of senior nursing staff at assessment, but not the seniority of the doctor, was associated with discharge.

**Clinical implications:**

Closer multiagency working is required as police are currently being recruited to fill a void between mental health services and the population they serve. Junior doctors require more senior support in making complex, and often risky, emergency management decisions with this population.

Place of safety legislation permits police officers to remove individuals thought to be mentally disordered and in need of immediate mental health assessment to a facility where this assessment can be carried out. Such legislation exists in many countries, including the UK,^1,2^ Germany,^3^ South Africa,^4^ Australia^5^ and the USA.^6^ Powers and legislation vary between and within countries but in general police must suspect a mental disorder *and* perceive significant, acute or serious risk of danger or harm to the individual concerned or to others in order to permit removal to a place of safety. In Scotland this power is detailed under section 297 of the Mental Health (Care and Treatment) (Scotland) Act 2003^1^ (this detention lasts for up to 24 h or until the individual has been assessed); similar powers in England and Wales are under section 136 of the Mental Health Act 1983.^2^

There have been efforts to reduce use of police stations as a place of safety, reduce overall use of the legislation and improve knowledge of the legislation.^7–9^ Practice, however, remains variable across Scotland^10^ and the rest of the UK.^11,12^ In Scotland the use of this legislation is rising, with 795 detentions in 2015–2016, up from 130 in 2006–2007.^10^

There is reasonable reporting of place of safety use and the characteristics of those subject to the legislation^1,5,13–15^ but little is known about the nature of police involvement and which factors, if any, are associated with hospital admission. This study in the Scottish Highlands aimed to characterise the nature of police involvement with those detained under place of safety legislation, the demographics of this population and aspects of the assessment process to determine factors that might be associated with admission.

## Method

We conducted a retrospective study by review of medical case records, including Place of Safety forms (POS1, v7.0) completed by police constables, of all individuals brought under place of safety legislation to New Craigs Hospital, Inverness in the NHS Highland region (the designated place of safety for the NHS Highland health board) between April 2016 and March 2017. All place of safety referrals were included in the project, including repeat detentions for any one individual. Individuals who were already in-patients when the place of safety legislation was applied were excluded from the analyses regarding admission to hospital.

Referrals were identified by collecting POS1 forms from the medical records department and cross-referencing with a place of safety referral record sheet in the intensive psychiatric care unit (IPCU), which is where most place of safety referrals are assessed. The junior doctor's assessment book and mental health assessment team's diary were also consulted to ensure that as many place of safety referrals as possible were identified. In an attempt to determine completeness of these records the Mental Welfare Commission for Scotland was contacted. Information about the assessment team's wider activity was gained from their team diary, which is updated reliably after each assessment. The POS1 form completed by police is not statutory and therefore any referral identified in case notes as being under place of safety legislation was included.

The POS1 form has an open text box to give details of the ‘circumstances giving rise to the removal of the aforementioned person to a place of safety.’ In all cases where a POS1 form was available this information was qualitatively analysed to characterise the nature of police involvement, the reasons for concerns and the reasons for the decisions made. Diagnosis was determined from clinical records and kept to broad ICD-10 diagnostic categories; more than one diagnosis was permitted, for example comorbid substance misuse and personality disorder. Diagnoses were often already established from clinical records rather than being based on the single urgent assessment.

Population data for calculating rates of referral are from census data from National Records of Scotland, a non-ministerial government department charged with collecting information about Scotland's people and history.^16^

The project was part of service development and audit and therefore did not require ethical approval.

### Statistical analysis

Statistical analyses were performed using Minitab 17.1.0 and SPSS 22.0 statistical software, both for Windows. The association between categorical and outcome variables was assessed using Pearson χ^2^-tests; where the cell count was low the likelihood-ratio chi-squared test was used. For continuous variables, Student's *t*-tests were performed. Given repeated referrals of some individuals, multivariable binary logistic mixed-effects regression was performed to predict admission using individuals as the random effect in order to account for individual-specific effect and control for the fixed effects of age, gender, diagnosis and distance from place of safety on detention. Robust estimation procedure was used to obtain the estimate of covariance matrix in order to ensure that model assumptions were met. Estimates of effects are presented as odds ratios (OR) and 95% confidence intervals (CI). The level of significance was set at *P* < 0.05.

## Results

We identified 237 place of safety referrals over the 1-year study period. Of these, 97% (*n* = 231) were recorded on the place of safety record form in the hospital IPCU and 99% (*n* = 234) had a completed POS1 form. The yearly referral rate under place of safety legislation for the health board was 74 per 100 000 population (NHS Highland serves about 320 000 people and is sparsely populated, covering an area of 32 500 km^2^: around a quarter of this population live in Inverness and surrounding areas). The referrals were for 185 different individuals, with 30 (16%) being brought on more than one occasion over the study period (the highest number was five referrals, which occurred for three individuals).

### Time variation in place of safety referrals

The rate of referral was similar throughout the year and did not vary by month (χ^2^ = 12.06, *P* = 0.359) or day of the week (χ^2^ = 4.63, *P* = 0.592). Detention under place of safety legislation was most likely to occur between 12.00 h and 17.00 h and least likely between 06.00 h and 12.00 h (χ^2^ = 48.81, *P* < 0.001), however no particular time of detention was associated with admission to hospital (χ^2^ = 6.99, *P* = 0.221).

### Characteristics and outcome of individuals referred

The characteristics and outcomes of place of safety referrals are detailed in Table 1 (analysis of 230 referrals). The admission rate overall was 33% (*n* = 75), with only 15% (*n* = 11) of those admitted being involuntarily under the Mental Health (Care and Treatment) (Scotland) Act 2003. The admission rate for those brought on place of safety legislation is not significantly different from the admission rate for those seen by the assessment team referred from other sources, i.e. referred by health professionals not police (χ^2^ = 0.41, *P* = 0.521); 79% (*n* = 182) of those referred had at least one previous contact with local mental health services.

**Table 1:** Characteristics and outcomes of place of safety referral

Measure	All^a^	Admitted^a^	Not admitted	*P*
Referrals, *n* (%)	230 (100)	75 (32)	155 (68)	–
Age, years: mean (s.d.)	35.5 (12.1)	36.5 (12.9)	34.9 (11.7)	0.408
Males, *n* (%)	151 (66)	43 (57)	108 (69)	0.065
Previously known to local services, *n* (%)	177 (77)	55 (73)	122 (79)	0.364
Assessed with senior nurse present, *n* (%)	198 (86)	58 (77)	140 (90)	0.026
Grade of assessing doctor, *n* (%)				
Foundation year 2	49 (21)	19 (25)	30 (19)	0.809
General practice trainee	75 (33)	23 (31)	52 (34)	
Core psychiatry trainee (year 1 or 2)	62 (27)	21 (28)	41 (26)	
Core psychiatry trainee (year 3) or above	44 (19)	12 (16)	32 (21)	
Diagnosis, broad ICD-10 category:^b^ *n* (%)				
10 – Substance use disorders	115 (50)	27 (36)	88 (57)	0.003
20 – Non-affective psychotic disorders	17 (7)	13 (17)	4 (3)	<0.001
30 – Affective disorders	9 (4)	6 (8)	3 (2)	0.033
40 – Anxiety and stress disorders	16 (7)	7 (9)	9 (6)	0.324
50 – Eating disorders	1 (0)	0 (0)	1 (1)	–
60 – Personality disorders	79 (34)	30 (40)	49 (32)	0.209
70 – Intellectual disability^c^	13 (6)	7 (9)	6 (4)	0.093
80 – Developmental disorders	0 (0)	0 (0)	0 (0)	–
‘No mental illness’/‘social stress’	45 (20)	3 (4)	42 (27)	<0.001
Approximate distance of detention from hospital,^a^^,^^d^ *n* (%)				
0–5 miles	154 (67)	42 (56)	112 (72)	0.018
5–10 miles	9 (4)	4 (5)	5 (3)	
10–15 miles	13 (6)	5 (6)	8 (5)	
15–20 miles	14 (6)	7 (9)	7 (5)	
>20 miles	13 (6)	9 (12)	4 (3)	

a. Excludes individuals who left the hospital during an in-patient stay and were returned by police under place of safety legislation.

b. Diagnosis obtained from assessment/discharge letters and kept in broad diagnostic categories, more than one diagnosis was permitted owing to frequent comorbidity, e.g. personality disorder and substance misuse.

c. Also known as learning disability in UK health services.

d. It was not possible to determine the location of detention in 12% (*n* = 27) of referrals from the Place of Safety (POS1) forms.

Where individuals were not admitted most were discharged to their own home or in care of friends/family (*n* = 136/155, 88%); in a small number of cases individuals were taken into police custody (*n* = 6, 4%) or transferred for medical care in the local general hospital (*n* = 7, 5%).

It is local policy for place of safety referral assessments, where possible, to be performed by two assessors. All place of safety referrals were seen by a doctor (of various grades but generally junior doctors in training up to CT3 grade; in only one case was assessment by a consultant) and most with a senior assessment nurse present (*n* = 198, 86%) – where senior assessment nurses were not available, doctors completed assessments with ward nursing or auxiliary staff or alone. There was a high degree of variability in admission rates, with a median of 29% (IQR = 39, range 0–100), but this was not influenced by the training grade of the doctor (χ^2^ = 1.60, *P* = 0.809). The presence of a senior assessment nurse was associated with patients not being admitted following assessment (χ^2^ = 4.98, *P* = 0.026).

Admission was less likely for individuals where it was thought difficulties were related to substance misuse (χ^2^ = 9.88, *P* = 0.003), social stress (χ^2^ = 14.18, *P* < 0.001) or if there was felt to be no evidence of mental illness (χ^2^ = 6.43, *P* = 0.011). A personality disorder diagnosis was proportionately more common in those admitted following assessment but not significantly so (χ^2^ = 1.57, *P* = 0.209). Diagnoses of non-affective psychotic disorders (χ^2^ = 16.07, *P* < 0.001) and affective disorders (χ^2^ = 4.55, *P* = 0.033) were more prevalent in individuals admitted following assessment.

Most of the place of safety referrals were from the local area, with 67% (*n* = 154) from within 5 miles of the hospital – this gives a yearly referral rate of 248 per 100 000 population for Inverness. The number of referrals reduces with increasing distance from the hospital but as distance from hospital increases admission is more likely (χ^2^ = 11.87, *P* = 0.018). The prevalence of substance misuse diagnoses reduces with increasing distance from the hospital (χ^2^ = 12.18, *P* = 0.016).

If individuals were admitted to hospital the median length of stay was 6 nights (IQR = 22, range 0–136). Significantly shorter admissions were seen for individuals who had a substance misuse diagnosis (χ^2^ = 11.32, *P* = 0.023). No other diagnoses were associated with the length of admission.

### Nature of police involvement from POS1 forms

**Fig. 1:**
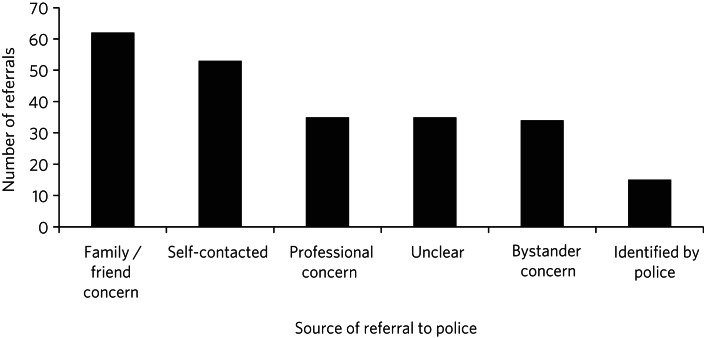
How police were alerted to individuals subsequently detained under the Mental Health (Care and Treatment) (Scotland) Act 2003 (total *n* = 234). Self-contact included presentation to a police station, telephoning police or approaching police in the street. Professional report was concern raised by health professionals or mental health support agencies such as Breathing Space. In some cases it was not clear from the Place of Safety (POS1) form details how the police had been alerted.

Police were alerted to individuals subsequently detained on place of safety legislation in a variety of ways (Fig. 1). Usually the alert came from concerned friends or family members (26%, *n* = 62/234), but it was also common for individuals to make themselves known to police (23%, *n* = 53), usually by telephone (15%, *n* = 34) but in some instances by walking into police stations (7%, *n* = 16).

The most common reason cited for police being involved was expression of suicidal ideation (73%, *n* = 167/230) (Table 2); 28% (*n* = 65) of individuals had made a gesture towards suicide (e.g. holding knife at their throat or being at a risky area on a bridge), and only 7% (*n* = 17) had made an attempt on their life (e.g. cut at their throat or removed from river after jumping in). Voicing suicidal ideation (χ^2^ = 23.58, *P* < 0.001), making a suicidal gesture (χ^2^ = 5.45, *P* = 0.020) and making an attempt on their life (χ^2^ = 4.43, *P* = 0.035) were all associated with not being admitted to hospital.

**Table 2:** Qualitative analysis of Place of Safety (POS1) forms for place of safety referrals^a^

Details recorded on POS1 forms	All^b^ (*n* = 230)	Admitted (*n* = 75)	Not admitted (*n* = 155)	*P*
Descriptors of harm to self, *n* (%)				
Voiced suicidal ideation	167 (73)	40 (53)	127 (82)	<0.001
Had made suicidal gesture	65 (28)	14 (19)	51 (33)	0.020
Had made suicide attempt	17 (7)	2 (3)	15 (10)	0.035
Voiced intent to self-harm	24 (10)	4 (5)	20 (13)	0.057
Had performed self-harm	17 (7)	2 (3)	15 (10)	0.035
Police noted previous suicide attempt	20 (9)	5 (7)	15 (10)	0.413
Other descriptors, *n* (%)				
Precipitating events, e.g. break-up/debts	28 (12)	4 (5)	24 (15)	0.027
Anxiety	4 (2)	2 (3)	2 (1)	0.454
Depression/low mood	16 (7)	3 (4)	13 (8)	0.220
Odd beliefs, perceptual disturbance, paranoia	34 (15)	18 (24)	16 (10)	0.006
Agitation/emotional/strange behaviours	38 (17)	17 (23)	21 (13)	0.081
Expressed intent to harm others	3 (1)	1 (1)	2 (1)	–
Location of detention under place of safety legislation, *n* (%)				
Public minor road	56 (24)	18 (24)	38 (25)	0.932
High-risk area, e.g. bridge/major road	39 (17)	12 (16)	27 (17)	0.788
Inside public building	31 (13)	12 (16)	19 (12)	0.436
Directly outside own home	21 (9)	9 (12)	12 (8)	0.293
Inside own home	15 (7)	2 (3)	13 (8)	0.100
Within hospital grounds	5 (2)	2 (3)	3 (2)	0.721
Rural area, e.g. woodland	6 (3)	1 (1)	5 (3)	0.371
Unclear	57 (25)	19 (25)	38 (25)	0.893

a. POS1 forms were available for 227 of the 230 referrals.

b. Excludes individuals who left the hospital during an in-patient stay and were returned by police under place of safety legislation.

In only a small proportion of referrals did police give information beyond that focusing on suicidality. Of note, there was infrequent recording of the events that led to the individual now experiencing difficulties. Where this was detailed it was usually brief and a social stressor (e.g. split from partner or financial concerns) – in cases where a stressor was identified (by police) individuals were usually not admitted (χ^2^ = 4.87, *P* = 0.027). Police did report details about odd beliefs, paranoia or perceptual disturbances (e.g. hearing voices or responding to unseen stimuli) in 34 referrals (15%) – this was associated with admission to hospital (χ^2^ = 7.51, *P* = 0.006) *and* with non-affective psychotic disorder diagnoses (χ^2^ = 4.85, *P* = 0.028). In the small number of referrals in which police described behaviours (outwith the suicidal acts/gestures), these descriptions were often very general and would not necessarily be related to mental ill health, for example ‘highly emotional’, ‘acting in a strange manner’, ‘agitated’, ‘shouting nonsense’, ‘unpredictable’ or ‘hysterical.’

Police were called to a range of areas in response to concerns for individuals subsequently detained under place of safety legislation (Table 2). The location from which individuals were removed was not associated with subsequent admission to hospital even when removal was from high-risk areas such as bridges or major roads (χ^2^ = 0.07, *P* = 0.788). In 7% of cases (*n* = 15/230) it appeared that individuals had been removed from their own home, which is not permitted under place of safety legislation in Scotland.^1^

### Multivariable binary logistic mixed-effects model

When controlled for age, gender and distance from the place of safety (i.e. the hospital) in a multivariable binary logistic mixed-effects regression with individuals as the random effect, the odds of admission were significantly higher for diagnosis of non-affective psychotic disorder (OR = 32.93, 95% CI 4.33–250.17, *P* = 0.001), affective disorder (OR = 15.13, 95% CI 2.15–106.61, *P* = 0.007), anxiety and stress disorder (OR = 7.01, 95% CI 1.21–40.93, *P* = 0.037), intellectual disability (OR = 17.58, 95% CI 2.32–130.02, *P* = 0.007) and personality disorder (OR = 5.49, 95% CI 1.21–24.86, *P* = 0.027) relative to those without a diagnosis of mental illness. Substance misuse was not significantly associated with admission (OR = 2.78, 95% CI 0.64–12.08, *P* = 0.170). Although not statistically significant, the individual's age was positively associated with admission and the odds of admission were higher for women than men. Also, odds of admission increased with distance from the place of safety – the farther the distance the higher the odds of admission. The use of individuals as the random effect ensures that extra-individual variations resulting from repeat referrals are adjusted for and this is evidenced in the size of the confidence intervals of the estimates.

## Discussion

Place of safety referrals constitute a significant proportion of urgent mental health assessments within the NHS Highland region – up to one-third of assessments within the study hospital. The recording of these referrals in the hospital appears to be reliable and numbers are in keeping with data from the Mental Welfare Commission for Scotland.^10^ NHS Highland accounts for perhaps up to 20% of all place of safety referrals in Scotland;^8^ this is proportionately lower than a local study 10 years ago, when up to 50% were in the NHS Highland health board.^7,8^ However, it is of note that in the current study almost 70% of place of safety referrals occurred within a few miles of the designated place of safety. This is unlikely to be simply due to population factors despite the place of safety being located in Inverness. The place of safety referral rate per year for Inverness is 248 per 100 000 – over 10 times the rate for Scotland as a whole and 3 times that for the health board with the highest referral rate in Scotland.^10^ Under-reporting in some Scottish health boards is likely to contribute to this variation but does not fully explain the vast differences. Looking more broadly, this referral rate is also significantly higher than in studies in England, where rates are reported between 59.8 per 100 000 in the North-East^12^ and 169 per 100 000 in Ipswich, Suffolk.^11^ There appears to be excessive use of this restrictive legislation in Inverness – factors driving this are likely to be poorer joint working, workload pressures and a lack of availability of alternative options of disposal/help for those in crisis. There are a striking number of referrals where individuals have sought help from police rather than from local mental health services. This supports the proposal in a recent Mental Welfare Commission for Scotland place of safety report that there is a gap between service provision and the needs of this distressed population.^10^

Police are responding to distressed individuals in a range of locations, from their own home to high-risk situations on major roads or high bridges. Where place of safety legislation is used, this is overwhelmingly in response to concerns about suicide risk. This study does show that police do well in identifying those in need of mental health support – the admission rate of police referrals is identical to that from other sources and they detail evidence of psychosis on their referrals. The language used by police in describing behaviours includes terms such as ‘highly emotional’ and ‘hysterical’ – these are commonly used terms but could be stigmatising for those with mental health difficulties. Targeted training and support for officers may well improve their interaction with distressed individuals and make involvement with police a less daunting experience for those with mental health problems.

The characteristics of those referred under place of safety legislation in the Scottish Highlands are similar to those reported elsewhere in terms of age, gender and outcome.^11,13,14^ However, in this study substance misuse problems are possibly more prevalent and were felt to be contributing to presentation in almost 50% of referrals whereas the proportion of severe mental illness is probably slightly lower.^6,14^ The compulsory admission rate is significantly lower (15%, compared with up to 50%) than in other reports, likely reflective of the lower proportion of individuals with severe mental illness.^11,13^

This study identified that presence of senior nurses influenced outcomes of assessments and recognised that there is significant variability in admission rates by doctors at all training grades. It is therefore important to ensure that experienced staff are conducting assessments and consideration should be given to a model that ensures multidisciplinary input such as that used in England and Wales,^2^ where approved mental health professionals (AMHPs) support the assessment process, including follow-up care arrangements for those not admitted. Further, with evidence in Scotland that trainees are doing fewer emergency assessments,^17,18^ ensuring that trainees are well supported by senior medical staff in completing these assessments is crucial.

### Predicting outcomes and length of hospital stay

Predicting outcome of assessments is challenging given the nature of any mental health crisis, as difficulties are very individualised. However, diagnosis appears to be a primary factor in the decision-making process. In general, where referrals are identified as being related to primarily social stressors or substance misuse problems admission to hospital is avoided or, where felt necessary, kept as short as possible. A diagnosis of severe mental illness was associated with admission but not any particular length of admission, and perhaps in some cases a short admission plays a containing role rather than being for treatment itself when individuals are presenting via police. Personality disorder diagnosis was associated with admission to hospital despite extensive local training, resource and a new integrated care pathway which states that admission for those with personality disorder is ‘at best neutral and at worst harmful’.^19^ It is likely that a combination of factors influence the decision to admit – those with personality disorder diagnoses may be in a high degree of crisis that cannot always be de-escalated over a single assessment and may be thought of as being at high acute risk of suicide, given their language, circumstances, social supports or expression of plans for suicide following assessment if they leave the place of safety. In addition, they may be displaying ‘pseudopsychotic’ phenomena that are felt to require admission for further assessment. In this context the seniority and experience of the assessing team is also likely to be important.

If individuals were detained further from the place of safety, admission was more likely – this is probably related to service provision in more rural areas, i.e. lack of crisis response teams as well as practicalities in supporting discharge to more rural areas in the evenings and overnight.

Expression of suicidality or self-harm in itself is not helpful in determining outcome, although it is an important factor in decision-making and risk assessment/management. Given that suicidal expression is actually associated with not being admitted, it may be that in such circumstances services could offer alternative interventions or supports (to police and individuals) to avoid use of legislation and the ‘frightening’ experience of being detained under place of safety legislation.^20^ Future studies exploring outcomes and service use in those with suicidality not admitted would be helpful in guiding service development and delivery.

### Strengths and limitations

This study has a lengthy period of data collection and a relatively large sample. This makes it similar to other studies in this field, which helps to allow any variation/patterns to be identified. Characteristics of the population are also similar to those reported elsewhere. This is the first study to analyse the nature of police involvement and link this with outcome of the mental health assessment. Efforts were made to ensure that all recognised place of safety referrals, especially those with completed POS1 forms, were identified over the study period – unfortunately, despite being contacted the Mental Welfare Commission for Scotland did not provide information to cross-reference those included in the project, which would strengthen the data-set. However, given the multiple sources and communications systems within the hospital and across the health board we are confident that the majority of place of safety referrals were identified. The study was carried out retrospectively, which limits the information available. Given the retrospective nature and use of a clinical cohort, diagnostic categories were kept broad and thus open to a degree of interpretation. However, the decisions and diagnoses made reflect day-to-day practice and thus findings are clinically informative.

### Improving practice

When individuals are brought by police to a place of safety it is important to complete an individualised assessment. Diagnosis is an important factor in the decision-making process and practitioners should remember that individuals not expressing suicidal ideation also have a significant mental health burden and may require admission. Experienced practitioners should be involved in multidisciplinary assessment where possible and it is crucial that junior medical staff are well supported by senior colleagues in making decisions.

Police are frequently responding to mental health crises and seem to do well in identifying those in need of urgent service contact. Training would likely improve knowledge and interaction between service users and police^21^ but is unlikely to have a major impact on the use of legislation or characteristics of those referred.^6,14,20^ Street triage services where police work more closely with mental health services (via telephone or in mobile units) are becoming established across the UK and are positively received by police officers.^22^ Recent systematic reviews highlight that there is a suggestion of positive outcomes such as reduced referrals and use of police jurisdiction^23^ but there remains limited robust evidence of efficacy and a lack of clarity on the best model for services.^22–24^

Ultimately this study identifies a breakdown between mental health services and those who require support, with police being recruited to fill the void. Joint working to improve awareness of and access to mental health services before crisis will be important in reducing use of restrictive legislation and improving outcomes.

## About the authors

**Struan Simpson** is a specialty doctor at the Phoenix Centre, Raigmore Hospital, Inverness, UK. **Jude Eze** is a quantitative scientist at the Epidemiology Research Unit (Inverness Campus), Scotland's Rural College (SRUC), Inverness, UK.
